# Mechanism Underlying Tissue Cryotherapy to Combat Obesity/Overweight: Triggering Thermogenesis

**DOI:** 10.1155/2018/5789647

**Published:** 2018-05-02

**Authors:** Suvaddhana Loap, Richard Lathe

**Affiliations:** ^1^SAS Clinic BioEsthetic, 11 Rue Eblé, 75007 Paris, France; ^2^Division of Infection and Pathway Medicine, University of Edinburgh, Little France, Edinburgh, UK

## Abstract

**Background:**

Local adipose tissue (AT) cooling is used to manage obesity and overweight, but the mechanism is unclear. The current view is that acute local cooling of AT induces adipocyte cell disruption and inflammation (“cryolipolysis”) that lead to adipocyte cell death, with loss of subcutaneous fat being recorded over a prolonged period of weeks/months. A contrasting view is that AT loss via targeted cryotherapy might be mediated by thermogenic fat metabolism without cell disruption.

**Methods:**

In this retrospective study of individuals presenting for cryotherapy to the Clinic BioEsthetic, Paris, France, we recorded waist circumference, body weight, and body mass index (BMI) by direct measurement and by whole-body dual-energy X-ray absorptiometric scanning. In select individuals, blood analysis of markers of inflammation and fat mobilization was performed before and after the procedure.

**Results:**

We report that (i) single sessions of tissue cryotherapy lead to significant loss of tissue volume in the time frame of hours and (ii) multiple daily procedures lead to a cumulative decline in AT, as assessed by waist circumference, body weight, and BMI, confirmed by whole-body dual-energy X-ray absorptiometric scanning. In addition, (iii) blood analysis following tissue cryotherapy found no significant changes in biochemical parameters including markers of inflammation. Moreover, (iv) calculations of heat extracted and of compensatory weight loss taking place through thermogenesis are substantially consistent with the observed loss of AT.

**Conclusions:**

These findings argue that cold-induced thermogenesis (“cryothermogenesis”) rather than adipocyte disruption underlies the reduction in AT volume, raising the prospect that more intensive cryotherapy may be a viable option for combating obesity and overweight.

## 1. Introduction

The growing prevalence of obesity and overweight is of increasing concern because deposition of subvisceral fat in obesity is associated with increased risk of life-threatening conditions including cardiovascular disease, diabetes, metabolic syndrome, and some forms of cancer [1–3].

Dietary interventions in obesity generally bring only short-term benefits, and attention has focused on alternative strategies for adipose tissue (AT) ablation including surgical removal and radiofrequency, ultrasound, and laser treatment [4, 5]. An alternative approach, body cooling, involves either environmental cold exposure (e.g., [6–9]) or devices to reduce the skin temperature (e.g., [10, 11]), leading to systemic or local exposure of fatty tissues to active cooling [12–15]. With respect to tissue cooling, it has been hypothesized that adipocytes are more sensitive to cooling than other tissue types and that cooling leads to crystallization of cytoplasmic lipids, disruption of cellular integrity, cell death via apoptosis/necrosis, and inflammation, leading to selective loss of AT over a period of weeks to months by a process dubbed “selective cryolysis” or “cryolipolysis” ([12, 16], reviewed in [14, 17]). Selective sensitivity of fatty tissue to cold has a long history, and for over a century there have been observations of local adipose tissue lesions in regions exposed to local cooling [15]. However, a competing hypothesis is that cold exposure might act by boosting energy expenditure via fat metabolism and thermogenesis (e.g., [18–20]), leading to reduction of fat mass without cell disruption. This possibility was long overlooked because it was thought that thermogenic adipose tissue, widely described in rodents, was not present in humans (see below).

Two principal types of AT have been described in mammals: white adipose tissue (WAT) and brown adipose tissue (BAT). The most abundant adipose cell types are the white adipocytes that contain a single intracellular lipid droplet and localize to specific depots within the body. White adipocytes store excess energy as lipid and function to regulate systemic energy balance through the release of adipokines that target peripheral tissues [21] and also target the brain to modulate appetite in response to excess energy supply [22, 23]. WAT expands by increasing the adipocyte size and/or number, and WAT expansion serves to protect tissues including the muscle and liver from lipotoxicity [24]. Depots of WAT are generally classified as visceral or subcutaneous, with the latter being considered to be protective, whereas the former are linked to metabolic disease [24–26].

The second major type of the adipose cell, the brown adipocyte, was overlooked in humans for many years until imaging with [^18^F]-fluorodeoxyglucose revealed that, like mice, humans also have extensive depots of BAT ([27], reviewed in [28, 29]). In contrast to WAT, BAT adipocytes express uncoupling protein (UCP1) that permits mitochondria to metabolize fat via *β*-oxidation to generate heat [29, 30]. Brown adipocytes are widely distributed in the supraclavicular and neck regions, with additional paravertebral, mediastinal, para-aortic, and suprarenal localizations [27, 29, 31], and oxidative metabolism in BAT has been demonstrated to contribute directly to increased energy expenditure in response to acute cold exposure in humans [32]. Importantly, numbers of brown adipocytes are thought to decline with age [8, 33], and this may contribute to increasing incidence of overweight with age.

BAT depots can expand in both metabolic rate and cell number to maintain the body temperature in response to cooling. By triggering BAT lipolysis and thermogenesis, cold exposure can reduce the body pool of fatty acids [19, 32], and prolonged cold exposure can induce the proliferation and differentiation of precursors, leading to an increase in brown adipocyte numbers [34–36]. There is also evidence that WAT can partly convert to BAT-like adipocytes in response to different stimuli [37, 38], suggesting that redirection of WAT precursors towards BAT (“browning”) in response to cold exposure could contribute to obesity control.

Systemic/environmental cold exposure has inconveniences as a modality for managing obesity/overweight, and attention has focused on the application of cold temperatures directly to fat deposits with a view to stimulating dissipation of lipid depots. FDA approval was given in 2010 for the use of a tissue cryotherapy device aimed at reducing abdominal fat; the safety and efficacy of the procedure have now been widely demonstrated (reviewed in [39, 40]).

Cooling of fatty tissue has been suggested to lead to adipocyte disruption/cell death and local inflammation (“selective cryolysis” or “cryolipolysis”) that precede AT loss [12, 16]. The mechanistic basis is of direct clinical relevance because if tissue cryotherapy operates by inducing adipocyte cell death, then repeat procedures must be widely spaced (e.g., 8 weeks apart) to allow removal of cell debris and resolution of inflammation [12, 39]. Indeed, it has been reported that beneficial results are only observed after weeks or months, the recommended area for application is limited to local prominences of fatty tissue (“saddlebags”), and it is necessary to wait for a long period (1–3 months) before proceeding to a new zone [12, 14, 16, 17]. By contrast, others have suggested the possibility that the beneficial effects of body cooling might take place by increasing nonshivering thermogenesis (e.g., [18, 19]); if confirmed, this would allow simultaneous application to different body regions, and moreover, repeated cryoexposure might be effective in driving the loss of adipose tissue [18].

In this retrospective study, we have addressed this issue (i) by studying changes in obesity-related parameters in response to single versus multiple/serial applications of tissue cryotherapy, (ii) by blood analysis for markers of inflammation and lipid mobilization, and (iii) by comparisons of profiles of weight loss against calculations of energy expenditure. We report that cryoexposure is not associated with biomarkers of inflammation and that serial daily applications lead to a cumulative reduction of waist circumference and BMI accompanied by AT reductions, confirmed by absorptiometric whole-body scanning. We also report that the extent of AT loss is consistent with the extent of loss predicted from calculated energy expenditure through compensatory fat metabolism and thermogenesis.

## 2. Methods

### 2.1. Subjects and Ethical Permissions

In this retrospective study, two groups of participants attending a medical clinic (SAS Clinic BioEsthetic) in Paris, France, in the period January 2016 to September 2016 were investigated: group 1, *n*=18 (three treatments), and group 2, *n*=7 (six treatments monitored by whole-body scanning) (summarized in Table 1). All had a BMI in either the normal range (67–71%) or overweight/obese (29–33%), and no subject undertaking the procedure was classified as underweight. Tissue cryotherapy, a noninvasive procedure [14, 17, 40, 41], does not require formal institutional ethical approval. All participants gave written informed consent for their involvement in the study, data analysis, and preparation for publication. Exclusion criteria were (i) allergic/inflammatory reaction to cold: a positive reaction (redness/inflammation, rash, itchiness, blistering, and any other adverse effects) on application of an ice cube for 2 minutes to the skin of the inner arm, or (ii) evidence/history of cryoglobulinemia and Hodgkin's or non-Hodgkin's lymphoma, including Waldenstrom macroglobulinemia.

### 2.2. Tissue Cryotherapy Procedure

The procedure employed a commercial tissue cryotherapy device (FG6601-006, ADSS, Republic of China) equipped with multiple cooling probes (cooling area per probe 20 × 8 cm). Weight, height, and waist and thigh (left) circumferences were recorded; BMI was calculated automatically using a commercial apparatus (model SC240MA, Tanita, Japan). A layer of wetted paper (glycerol-based wetting membrane, ETG-111-200, Freezefats, Republic of China) was applied to the lower back and hips of the reclining subject, followed by symmetrical application of six probes placed pairwise accompanied by gentle suction to improve contact. The cooling temperature was set to −10°C, declining to −5°C over 30 minutes; the application duration was 40 minutes. Subjects then lay supine, and the procedure was repeated on fatty deposits on the lower abdomen and hips, again for 40 minutes (total duration 1.33 h). Oral (sublingual) temperature, a proxy for core temperature, was recorded before and at 1, 5, 10, and 30 minutes after the procedure. Local skin surface temperature at the site of treatment was recorded using a laser thermometer 1, 5, 10, 30, and 60 minutes after removal of the cooling probe. Body weight, waist and thigh circumferences, and BMI were recorded again within 1 h after the procedure. Group 1 participants underwent three procedures; group 2 participants volunteered for multiple procedures and agreed to undergo whole-body scanning before and after three and six sequential procedures.

### 2.3. Whole-Body Scanning

Whole-body assessment of fat content employed a Lunar iDXA dual-energy X-ray absorptiometry scanner (GE Healthcare Lunar, Madison, WI, USA) in which the subjects lay supine; the maximum scan time was 7.2 minutes. Scanning and recording of results were according to the manufacturer's instructions.

### 2.4. Analysis of Blood Profiles

Seven subjects provided a comprehensive test battery of blood analyses before and 3 days after receiving tissue cryotherapy. Blood analyses were performed by a certified bioanalytical laboratory (Laboratoire Philippe Auguste, Paris, France) and included markers of (i) inflammation (monocyte counts, C-reactive protein, and neopterin, a marker of macrophage/dendritic cell activation); (ii) insulin resistance (IR) and homeostasis model assessment (HOMA) test (insulin-glucose balance); (iii) lipid profile (total cholesterol (TC) and high-density lipoprotein (HDL)) including markers of hepatic steatosis and fat metabolism (alanine aminotransferase (ALAT), alkaline phosphatase (ALP), aspartate aminotransferase (AST), gamma-glutamyl transferase (GGT), triglyceride, (TG), and their relevant ratios); and (iv) thyroid hormone (TSH, total T4, and free T4).

### 2.5. Precision/Variability

To assess the precision/variability of individual measures of weight, BMI, fat mass, and waist and thigh circumferences and to exclude this as a significant complicating factor, a random sample of individuals (*n*=12) from group 1 were subjected to three to five repeat measures of these parameters at the same time by different operators, and the standard deviations were calculated: repeat measure SDs were weight 0.032 kg (0.046%), BMI 0.002 units (0.111%), waist circumference 0.76 cm (0.9%), and thigh circumference 0.79 cm (1.470%); all with the exception of thigh circumference were smaller than the changes observed following the procedure, validating statistical analysis.

### 2.6. Statistical Analysis

In this retrospective study, there was a tendency for subjects with lower indices of obesity to opt for more frequent applications of the procedure (not presented), and to avoid bias in statistical comparisons, all values were expressed as percentages of the baseline. Assessment of statistical significance employed normalized means and SDs; results were evaluated using unpaired Student's *t*-tests, and the statistical significance was set at *p* ≤ 0.05.

## 3. Results

### 3.1. Local Cooling Produces Decrements in Waist Circumference

**Figure 1:**
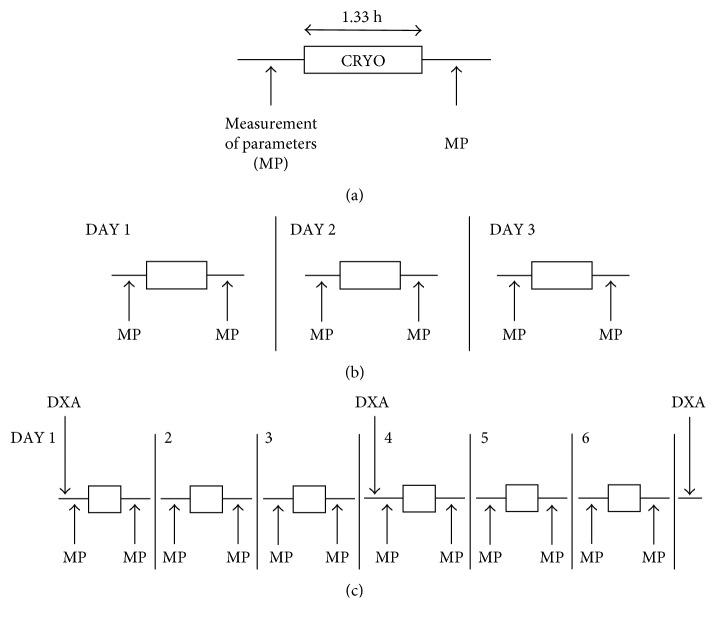
Timeline of the cryotherapy procedure. (a) Standard procedure in which physiological parameters (weight, BMI, fat mass, and waist and thigh circumferences) are measured (MP) before and after cryotherapy (CRYO, duration = 1.33 h) in all procedures (not to scale in (b) and (c)). (b) Group 1 (*n*=18) underwent the procedure on 3 serial days. (c) Group 2 (*n*=7) underwent the procedure on 6 serial days; dual-energy X-ray absorptiometry (DXA) scanning was performed as indicated before the procedure and following three and six procedures.

The cryotherapy procedure (summary timelines are given in Figure 1) was applied to a first series of 18 subjects (group 1), and waist circumference, body weight, BMI, and local and oral (sublingual) temperatures were recorded before and after a single application of local abdominal tissue cooling. Local skin surface temperature immediately beneath the cooling probe was variable within the region but did not fall below +6°C. There was typically local reddening of the skin consistent with increased local blood circulation. No superficial skin damage of any type was observed, there was no evidence of tissue “solidification,” no shivering thermogenesis was observed, and no participant reported any adverse events.

Because indices such as waist and limb circumferences are to some extent subjective measures, we carefully examined the variability/precision of the values. There was some repeat measure variability, but the extent of intermeasure variability was generally less than the documented changes (for details, see Methods), permitting statistical analysis. In addition, there were no statistically significant differences in the responses of male and female subjects (not presented), and all analyses addressed the means and SDs across the entire group.

**Figure 2:**
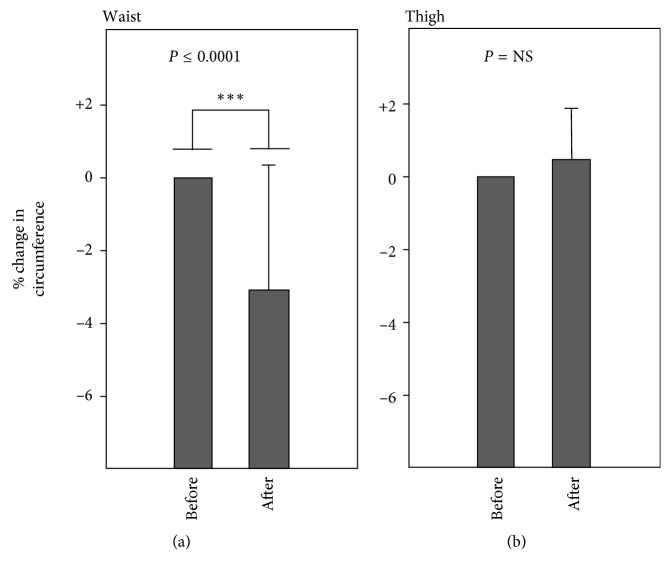
Percentage change in (a) waist circumference and (b) thigh circumference, following single abdominal applications of tissue cryotherapy (group 1; duration of the procedure, ca. 1.33 h; time following the procedure, ca. 1 h). Error bars indicate SD; error bars are not shown for the left-hand plots in each panel because these were normalized to 100%.

As shown in Figure 2(a), there was a mean reduction of 3.0% of the waist circumference (mean 2.55 cm; *p* < 0.0001) within 3 h following the procedure, but there was no significant change in thigh circumference (Figure 2(b)). The body temperature (oral) rose from mean 35.8 to mean 36.1 (+0.3°C), but the change was not significant; changes in weight and BMI on single applications also fell short of statistical significance (not presented; see below). There was a small trend towards a reduction in cryotreatment-induced weight and BMI changes as a function of age, although the trend was not statistically significant (*p*≅0.2): some older individuals displayed substantial reductions in waist circumference and some young subjects displayed low responses (not presented).

To address a possible delay/time-dependent effect, measurements were performed 5–10 days after the procedure but without further tissue cryotherapy. The extent of the reduction in waist circumference (∼1 week after cryotherapy; mean 3.3 cm; 3.9%; SD = 3.09 as percent) did not achieve statistical significance versus the reduction observed immediately following a single procedure (mean 2.55 cm; 3.0%; SD = 2.71 as percent; not presented), although there was a trend towards further reduction.

### 3.2. Multiple Applications Produce Incremental Loss of Weight and BMI

**Figure 3:**
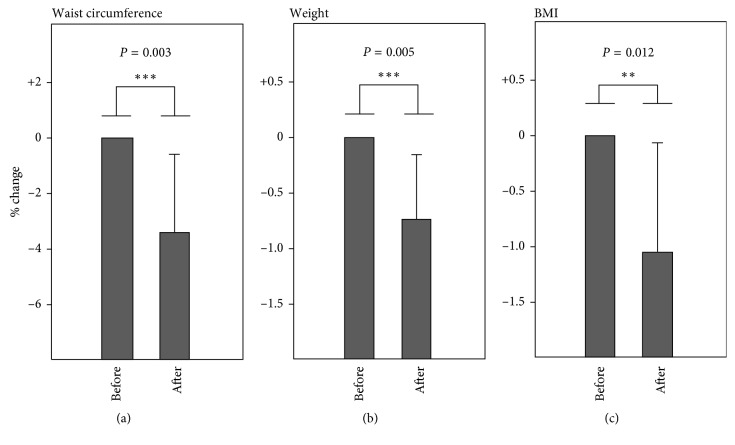
Decrement in (a) waist circumference, (b) weight, and (c) BMI following three sequential daily applications of tissue cryotherapy. Error bars indicate SD; error bars are not shown for the left-hand plots in each panel because these were normalized to 100%.

We then investigated whether serial daily applications of tissue cryotherapy might produce greater losses of AT. Group 1 subjects undertook subsequent procedures on days 2 and 3 under the same conditions and for the same duration as on day 1. As shown in Figure 3, in addition to a significant (*p*=0.003) decline in waist circumference (mean 2.8 cm; 3.3%), significant reductions in both body weight (mean 0.53 kg; 0.73%; *p*=0.005) and BMI (0.2 units; 1.1%; *p*=0.012) were recorded following three procedures (Figure 3).

### 3.3. Whole-Body Scanning Confirms AT Loss

**Figure 4:**
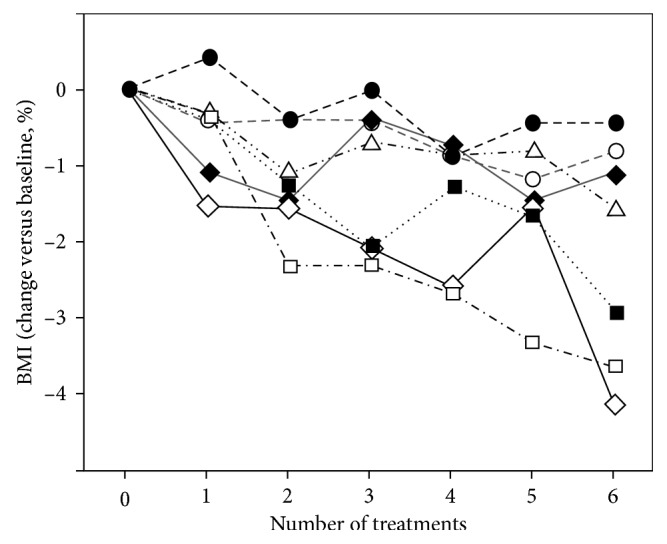
Progressive decline in BMI (expressed as %) in group 2 undergoing six rounds of tissue cryotherapy. Although all individuals responded, the figure illustrates the interindividual variability, in accord with other studies (e.g., [18, 32]).

**Figure 5:**
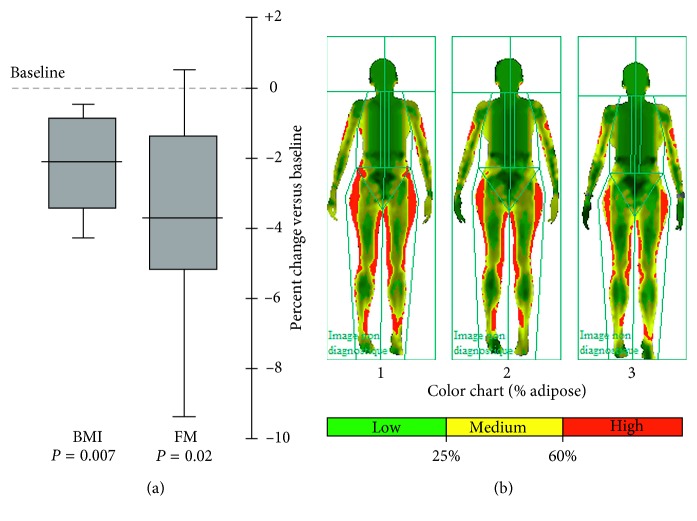
Decline in BMI and fat mass (FM) with six treatments. (a) The box-and-whisker plot (median, quartiles, range) of BMI and computer-generated FM% values by whole-body dual-energy X-ray absorptiometry scanning in group 2 before (baseline) and after six rounds of the tissue cooling procedure. (b) A representative example of the output of the dual-energy X-ray absorptiometry body scanner in one subject before the procedure (1), after three procedures (2), and after six procedures (3).

To rigorously confirm changes in body mass composition, individuals in group 2 volunteered for independent evaluation via whole-body dual-energy X-ray absorptiometry and computerized calculation of body fat mass content (%) before and after three procedures and again following six procedures; BMI in these subjects was measured as before. Significant declines in both parameters were seen following three applications (not presented), and, following six applications, mean scanning-based fat mass was reduced by ∼3.8% (*p*=0.02 versus baseline, paralleling the reduction in independently assessed BMI (∼2.1%; 0.42 units; *p*=0.007 versus baseline; Figure 4 and 5). Dual-energy X-ray absorptiometry thus accords with other independent measures of adipose content (waist circumference, body weight, and BMI) and confirms that serial tissue cooling procedures lead to a progressive reduction of body fat content.

### 3.4. Biomedical Parameters

To assess whether local tissue cooling is associated with adipocyte disruption, blood analysis of markers of inflammation and lipid mobilization (as given in Methods) was carried out on seven randomly selected subjects before and 3 days after six serial daily procedures (Supplementary Materials S1). This timepoint was chosen to maximize the likelihood of detectable systemic changes because fat “freezing” and resolution, implied to underlie cryolipolysis, are likely to take days or more to resolve. One subject displayed an elevated HOMA-IR value (5.16) before the procedure, but this normalized at follow-up after treatment (1.19; Supplementary Materials S1), and in one subject there was an elevation of neopterin for reasons unknown. As expected, there was a nonsignificant trend for all treated subjects who are overweight/obese to have an elevated HOMA-IR score (Supplementary Materials S1). All other profiles before and after the procedure were unremarkable. Importantly (except as noted above), there was no indication of inflammation or abnormal lipid profiles. Tissue cryotherapy in these subjects was thus not associated with systemic inflammation or changes in blood lipid profiles that would be consistent with ongoing cell lysis/adipocyte disruption.

## 4. Discussion

We report that serial daily abdominal tissue cooling (tissue cryotherapy) produces progressive loss of AT, confirmed by dual-energy X-ray absorptiometry. No adverse events were reported for the participants in this study, in accordance with previous studies and meta-analysis that the tissue cryotherapy procedure is safe and well tolerated [14, 17, 40, 41]. Following six procedures, there was a parallel decline in BMI (∼2%; 0.5 units) and fat mass content determined by whole-body scanning (∼3.8%). Under carefully controlled conditions, no consistent changes in biochemical parameters or adverse events were reported following six procedures, and there was no evidence of systemic inflammation or abnormal fat mobilization indicative of disruption of cellular integrity and cell death via apoptosis/necrosis (“cryolipolysis”).

Our findings cast light on the mechanism of AT loss. It has been suggested that the presence of fatty deposits renders adipose cells highly susceptible to cell killing produced by cooling, mediated by aggregation/crystallization mechanisms leading to cell disruption and/or apoptotic/necrotic cell death, followed by local inflammation and a protracted period (weeks) of immune cell infiltration and clearance of cell debris [12, 14, 16, 17, 39]. However, in contrast to earlier reports that there is no evident change in body fat shortly after the procedure [14, 16, 17, 39], we report significant beneficial effects within a short time frame after the procedure, an effect too rapid to be explained by cell death and clearance. In addition, serial daily applications of tissue cooling produced further progressive losses of waist circumference, body weight, BMI, and body fat mass that could be incompatible with the cell disruption hypothesis. Furthermore, biochemical analysis revealed no adverse changes, in accordance with a previous report that also found no significant changes in blood parameters following cooling applied using a liquid-conditioned tube suit at 18°C [32]. The lack of markers of inflammation or lipid mobilization in subjects undertaking the procedure argues against the adipocyte disruption hypothesis and instead points to adipocyte cell volume reduction via thermogenesis, with conservation of cellular integrity. In support, the body temperature (oral) was maintained (+0.3°C) despite intense local cooling, suggesting that thermogenesis is taking place.

Rigorous demonstration that thermogenesis is taking place will require invasive histological analysis of markers of thermogenesis (e.g., *UCP1* mRNA) and/or radiotracing of lipid mobilization and oxidation, which was not possible in this retrospective analysis. We therefore used a different approach to address whether thermogenesis in response to heat loss could underlie the observed decline in fatty tissue. We studied the relationship between measured physiological parameters and calculated measures of heat extraction and compensatory thermogenesis. First, using a simplified model of abdominal obesity (Supplementary Materials S2), the mean decline of circumference following three procedures (3.3%) was estimated to equate to a tissue loss of 0.50 kg, close to the observed mean decline of 0.53 kg, pointing to internal consistency of these independently measured values.

Second, data supplied by the manufacturer of the cryotherapy machine, revised according to direct measurements in a model system (Supplementary Materials S3), allowed us to calculate that, over a single session, the total heat extracted is in the vicinity of 1330 kcal. In the absence of compensatory thermogenesis, this would lead to a calculated mean fall in a body temperature of 18°C (Supplementary Materials S3), whereas no decline was observed (instead there was a small increase, +0.3°C; see earlier), indicating that thermogenesis is taking place.

Third, we compared the calculated quantity of heat extracted against the extent of tissue loss expected via compensatory thermogenesis. After three sessions (ca. 3990 kcal extracted), at a consensus value of 7710 kcal per kg fatty tissue metabolized (Supplementary Materials S3), the expected mean weight loss is 0.52 kg, compared with the observed weight loss of 0.54 kg. The mean estimated weight loss taking place via thermogenesis alone (calculated from heat extracted; no change in the body temperature) is therefore close to the actual weight loss. This broad equivalence between fat mass loss and energy expenditure substantiates, in accordance with previous reports [18, 19, 32], that energy expenditure taking place via cold-induced fat metabolism and thermogenesis may alone explain the beneficial effects of cold treatment.

Heat generation in response to cooling has generally been attributed to fatty acid *β*-oxidation. Exposure of mammalian cells to low temperatures (“cold shock”) causes widespread changes in gene expression (reviewed in [42, 43]). There is strong evidence that fat cells can directly sense low temperature and activate thermogenesis [20]; cold-induced upregulation of the transcription factor Zfp516 induces the expression of UCP1 [44], and BAT metabolic activity increases very rapidly following cold exposure [45]. Indeed, cold exposure induces rapid triglyceride uptake from the blood [30], and adipose triglyceride lipase activity is instrumental in BAT fat mobilization [46, 47]. Nevertheless, the exclusive emphasis on fatty acid oxidation by BAT may not be correct. It has been reported that, in human cold exposure, lipid oxidation only increased by 63%, whereas carbohydrate oxidation increased by 588% [48]. BAT contains significant endogenous glycogen stores, and cold exposure in rats and mice increases BAT glucose uptake by an order of magnitude [49, 50]. Glycolysis could therefore make a major contribution to increased energy expenditure during cold exposure, although short-term energy expenditure via glycolysis may well subsequently equilibrate via longer-term cellular fat metabolism (see below). Nevertheless, although increased BAT uptake of both glucose and free fatty acids was reported in response to body cooling, it has been argued that triglyceride metabolism represents the primary energy source for cold-induced thermogenesis [32].

Despite the focus on BAT, there is evidence that WAT may also contribute to cold-induced thermogenesis. Yoneshiro et al. reported that only around 50% of subjects displayed cold-activated BAT. Despite this, energy expenditure following cold exposure in their study also increased in subjects classified as BAT negative [18], indicating that non-BAT tissues may contribute. Because cold exposure does not appear to increase muscle energy metabolism [51], cold-induced thermogenesis in WAT could potentially explain the induced energy expenditure in BAT-negative subjects. Furthermore, both WAT and BAT are capable of metabolizing energy by pathways independent of UCP1 [52]; it is therefore possible that both WAT and BAT contribute to thermogenesis in response to tissue cooling. We note that there may be two components to cold-induced AT loss, involving (i) rapid energy expenditure by WAT and/or BAT, followed by (ii) slow AT loss as a result of continued metabolic activity, possibly including replenishment of glycogen stores via fat metabolism and, in the longer term, potentially facilitated by adaptive conversion of WAT to BAT (“browning”). In support, although falling short of statistical significance, in our study, there was a trend towards continued AT loss over the days following single procedures. We also observed that, in a small number of subjects who were followed up for up to 3 months following multiple serial procedures, significant AT loss continued to take place for an extended period (Supplementary Materials S4). Nevertheless, it was not possible to supervise these subjects for potential changes in activity/exercise and/or caloric intake during the follow-up period, and continuing changes therefore may not be unambiguously ascribed to metabolic changes induced by the cryotherapy procedure.

Regarding the safety of the tissue cryotherapy procedure, no consistent changes in biochemical parameters were observed following tissue cooling, including markers of inflammation. No adverse effects were reported by the participants, confirming previous reports regarding the safety and efficacy of the procedure [14, 17, 39–41]. However, there has been discussion about the possibility that, in mice, prolonged exposure to subthermoneutral temperatures might predispose to immune system deficiency (e.g., [53]) and could potentially increase the risk of infection or cancer. However, mice exposed to subthermoneutral temperatures are chronically maintained under these conditions for months to years. We note that, in humans, the induction of profound core hypothermia is an accepted medical procedure in both children and adults for the treatment of traumatic brain injury [54–56]. Tissue cryotherapy, by contrast, is an acute local procedure, and we observed no change in the body temperature (oral; mean change after the procedure, +0.3°C; *p*=NS). We surmise that the benefits of combating obesity, a major health risk, are likely to substantially exceed the potential risks, if any, of short-term local tissue exposure to reduced temperatures. Moreover, the benefits of cryotherapy may not be restricted to combating obesity: in addition to reducing measures of obesity through cold-induced fat metabolism, cold exposure can have further beneficial effects such as promoting HDL turnover and reverse cholesterol transport, with likely protective effects against atherosclerosis and heart disease [47].

Our findings raise a further issue of interest. Does local tissue cooling cause AT reduction principally in the cooled tissue (as predicted by the cryolipolysis theory), or is there a systemic reduction? Following a single procedure, no changes were observed at a site not exposed to local cooling, but whole-body scanning indicated significant AT losses at nonexposed sites after three and six sequential procedures (Figure 5, see also Supplementary Materials S4), indicating that systemic changes can take place after a longer time period. In addition to systemic depletion of fatty acid levels by BAT metabolism, there are direct neuronal pathways between AT and the hypothalamus, leading to activation of the sympathetic nervous system and the release of noradrenaline (NA) that promotes BAT activity (reviewed in [57]). A further contender is that systemic AT loss may be mediated by cytokines released from the cooled tissue, such as fibroblast growth factor type 21 and interleukin-6 (reviewed in [23, 58]), that might act both centrally (e.g., via the hypothalamus) and peripherally to modulate systemic fat metabolism (reviewed in [29]). In support, BAT transplantation in mice can induce systemic changes in the recipient ([58] for review). Behavioral changes (e.g., activity and food consumption) potentially mediated by endocrine targeting of the hypothalamus and/or hippocampus [59] may also take place, and further studies on the complex neuroendocrine interplay between body temperature, BAT and WAT fat metabolism, and adaptive behavior are warranted.

In summary, this work addresses the competing cryolipolysis versus cryothermogenesis interpretations of the mechanism underlying tissue cooling as a remedial therapy for overweight/obesity. In agreement with the previous work, local cooling of abdominal fatty tissue significantly reduced the measures of obesity, including waist circumference, body weight, BMI, and fat content. Our central observations are that (i) repeat procedures at short timescales produce progressive losses of AT, a finding inconsistent with “cryolipolysis” that is inferred to require weeks or longer between sequential treatments; (ii) blood profiling after the tissue cooling procedure gave no evidence of markers of inflammation or cell disruption; and (iii) calculated weight loss through thermogenesis alone was substantially consistent with estimates of heat extracted versus compensatory heat generation through enhanced tissue metabolism and thermogenesis. Our findings indicate that cold-induced thermogenesis (cryothermogenesis) rather than adipose tissue disruption is likely to underlie the observed reductions in measures of obesity following local tissue cooling.

## Figures and Tables

**Table 1:** Subject group parameters.

	Minimum	Maximum	Mean
Group 1 (*n*=18)			
M/F			*M* = 22%
Age (years)	19	82	54.3
Waist (cm)	70.1	112.5	85
Weight (kg)	55.8	96.4	71.7
BMI	18.9	32.7	24.8
Underweight BMI, <18.5			0%
Normal range BMI, <25			67%
Overweight BMI, 25–30			22%
Obese BMI, >30			11%
Group 2 (*n*=7)			
M/F			*M* = 14%
Age (years)	19	70	53.6
Waist (cm)	75.5	90	82.9
Weight (kg)	50.3	72.5	67.5
BMI	19.2	29.9	24.2
Underweight BMI, <18.5			0%
Normal range BMI, <25			71%
Overweight BMI, 25–30			29%
Obese BMI, >30			0%

## Supplementary Materials

Supplementary Materials S1: biomedical parameters. S2: consistency of weight loss versus physical dimensions. S3: consistency of energy extracted and weight loss. S4: evidence for long-term changes following cryotherapy.
