# Supplementary material for: Mechanism Underlying Tissue Cryotherapy to Combat Obesity/Overweight: Triggering Thermogenesis
Source: J Obes. 2018 May 2;2018:5789647. doi: 10.1155/2018/5789647 (PMC5954866; doi:10.1155/2018/5789647)
Supplement: Supplementary Materials — S1: biomedical parameters. S2: consistency of weight loss versus physical dimensions. S3: consistency of energy extracted and weight loss. S4: evidence for long-term changes following cryotherapy. [file 5789647.f1.pdf]

Supplementary data online

Mechanism underlying tissue cryotherapy to combat obesity/overweight – triggering thermogenesis

S. Loap and R. Lathe

S1. Biomedical parameters

S2. Consistency of weight loss versus physical dimensions

S3. Consistence of energy extracted and weight loss

S4. Evidence for long-term changes following cryotherapy

S1. Biomedical Parameters

		Parameter and units (reference range)[a]															
		Monocytes/mm3	CRP (mg/l)	NEO (nmol/L)	TC (mmol/L)	Chol-HDL (mmol/L)	TC/Chol-HDL	TG (mmol/L)	ALAT (U/L)	ASAT (U/L)	GGT (U/L)	PAL (U/L)	TSH (mUI/L)	Free T3 (pmol/L)	Free T4 (pmol/L)	INS (µUI/mL)	HOMA[b]
	Before / after	(240-820)	(<5)	(<7)	(<5.18)	(0.78-2.20)	(2.5-3.5	(<1.70)	(<34)	(<31)	(<38)	(42-98)	(0.30-3.60)	(3.38-6.45)	(102.96-218.79)	(2.6-24.9)	(0.74-2.26)
P001	B	340	<5	9	4.53	1.48	3.07	1.23	18	18	22	56	2.01	4.19	128.06	4.79	1.12
P001	A	500	<1	6.5	4.61	1.68	2.74	0.61	20	19	18	56	3.88	4.33	146.72	5.94	1.42
P002	B	750	<1	2.4	5.88	2.38	2.47	0.81	12	11	20	36	0.97	4.61	204.76	7.79	1.33
P002	A	530	<1	23.2[c]	4.66	2.15	2.17	0.75	10	12	18	31	1.61	4.42	170.40	4.43	0.84
P003	B	400	<1	ND	4.29	1.55	2.77	1.22	64	63	29	ND	3.41	4.45	144.79	2.21	0
P003	A	360	<5	2.2	5.61	1.74	3.24	1.27	66	63	32	78	4.09	4.24	131.66	2.52	0.52
P004	B	580	<5	ND	4.97	1.22	4.09	0.95	15	16	20	ND	ND	ND	ND	18.86	5.16[d]
P004	A	610	2	ND	4.63	1.04	4.48	0.88	21	26	18	81	3.38	4.53	189.83	5.53	1.19
P005	B	440	<1	ND	3.34	1.32	2.53	0.84	35	24	ND	ND	ND	ND	ND	5.14	ND
P005	A	ND	ND	ND	3.50	1.30	2.70	1.07	36	23	ND	ND	ND	ND	ND	ND	ND
P006	B	550	<1	3.3	4.87	1.40	3.48	1.21	29	22	28	ND	ND	ND	ND	13.67	3.2
P006	A	ND	ND	8	4.30	1.50	2.86	1.15	29	23	ND	ND	0.6	5.30	190.35	ND	ND
P007	B	450	3	5	ND	ND	ND	ND	17	22	48	51	3.83	3.79	144.79	6.34	1.59
P007	A	420	<5	<1	4.87	1.45	3.36	0.79	13	17	27	69	ND	ND	ND	ND	ND

[a]Abbreviations: A, after the procedure; ALAT, alanine aminotransferase (U/L); ASAT, aspartate aminotransferase (U/L); B, before the procedure; CRP, C reactive protein (mg/L); GGT, gamma-glutamyl transferase (U/L); Chol-HDL, cholesterol-high-density lipoprotein (mmol/L); HOMA, insulin resistance and homeostasis model assessment; ID, subject number; INS, insulin (microU/ml); ND, not determined; NEO, neopterin (nmol/L); PAL, alkaline phosphatase (U/L); TC, total cholesterol (mmol/L); TG, triglycerides (mmol/L); T3/T4, thyroid hormone (pmol/L); TSH, thyroid stimulating hormone (mU/L).

[b]Threshold HOMA-IR values range from 1.8 to 3.8 depending on the study [1]; typical accepted values are: healthy insulin sensitivity, 0.4–1.4; early insulin resistance, >1.9; significant insulin resistance, >2.9 (e.g., https://www.thebloodcode.com/homa-ir-calculator/).

[c]One subject displayed an elevated neopterin value.

[d]One subject displayed an elevated HOMA-IR value before the procedure, but this normalized after the procedure.

S2. Physical Dimensions and Weight Loss

In the main paper it was estimated that waist circumference declined by ~3.3% after three rounds of the cryotherapy procedure. At the same time weight loss was calculated at ~0.73%, against mean weight before treatment = 72 kg, and mean weight loss = 0.53 kg.

To assess whether these values are internally consistent we generated a simple model. In juveniles and young adults adipose tissues are widely distributed. By contrast, with age, adiposity tends to increase in specific areas. Although these differ between individuals, a common zone predominates in the abdominal area of both males and females. In a simple model the abdomen was considered as a ring with diameter 85 cm (subject mean circumference before treatment), with height 15 cm.

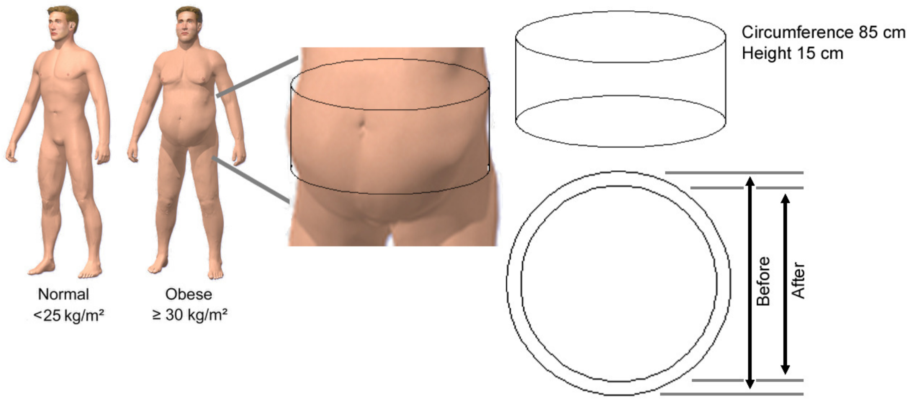

Images at left reproduced with permission from Wikipedia (https://en.wikipedia.org/wiki/Obesity)

Fatty tissue has a density of about 0.9 g/ml, the mass of this total volume
= 575.0 cm^2 x 15 cm x 0.9 = 7.729 kg

Following a 3.3% reduction in circumference the area is 537.6 cm^2, a reduction of 6.5% in both area and cylindric volume.

Calculated total loss of tissue = 0.065 x 7.729 = 0.503 kg

Observed weight loss, versus weight loss calculated from waist circumference, following three treatments

	**Weight loss**
Observed	0.53 kg
Calculated from physical dimensions	0.50 kg

Although the model is highly simplified, the equivalence of these values indicates that the loss of waist circumference is broadly consistent with the observed weight loss.

S3. Cryotherapy: Energy Extracted and Weight Loss – Are the Values Consistent?

(i) Weight Loss Equivalents in Kilocalories (kCal) Energy Expenditure

The generally accepted conversion factor is 3500 kCal per pound (454 g) of weight loss [2]. This figure has been questioned [3], but although it may overestimate weight loss in lean individuals, it is thought to be accurate in overweight/obese [4]. Accordingly, in the following analysis the value of 3500 kCal/lb, which equals 7710 kCal/kg, is employed.

(ii) Heat Extraction

Zelickson and colleagues have estimated the heat extraction according to the cooling intensity factor (CIF). In pigs (low dose) this was estimated at 21.5, equating to −36.8 W/cm^2 (incorrectly stated as mW/cm^2) [5]. The Zeltiq prototype device employs a lower CIF of −6 to −10 W/cm^2 [6].

In the main paper the size of the cryoprobe cooled area is 16 x 4 cm, and with 6 probes the total area exposed is 384 cm^2. At a CIF of 10 W/cm^2 this equates to 3840 W of energy extracted. Using an online converter (http://www.unitconversion.org/power/watts-to-kilocalories-it--per-hour-conversion.html) 5107 W would be 3301 KCal/h.

However, because the equipment employed in the main paper differs from that used by Zelickson and colleagues, we measured the rate of heat extraction directly in a model (water-filled balloon) designed to simulate the abdomen by measuring the change in water temperature (starting temperature = 35°C) as a function of time, that gave a lower value of 172 kCal/h per probe, approximating to 1000 kCal/h, indicating a lower CIF for this device.

(iii) Calculation of Weight Loss

At a rate (revised) of 1000 KCal/h, with each session lasting 1.33 hours, and a total of three sessions, the total heat extracted = 3990 kCal. If this quantity of heat is replaced by thermogenesis based on adipose tissue, at 7701 kCal/kg, the expected weight loss = 0.52 kg

Observed and calculated weight loss from heat extracted

	**Weight loss**
Observed	0.54 kg
Calculated loss from thermogenesis	0.52 kg

Allowing for experimental error, potential inaccuracies in the kCal/kg conversion values, and other complexities (e.g., the model does not account for vasoconstriction in the cooled tissue) these two values are broadly consistent.

(iv) Calculated Changes in Body Temperature Without Thermogenesis

Cryotherapy involves heat extraction, calculated here at 1000 KCal/h. With mean body weight 72 kg, treatment lasting 1.33 h, a reduction in body temperature of ~18°C is calculated, to be contrasted against the observed small (but not significant, $P > 0.05$) core temperature increase (+0.3°C), indicating that thermogenesis is taking place.

S4. Long-Term Changes Following Cryotherapy

A small number of subjects (not presented in the main paper) were followed for an extended period following the cryotherapy procedure, and provided evidence of ongoing loss of fatty tissue. In the example given (Figure below), one subject received five sequential applications of the procedure, and was examined by dual X-ray absorptiometric scanning before the cryotherapy procedure and then 14 and 60 days after the procedures.

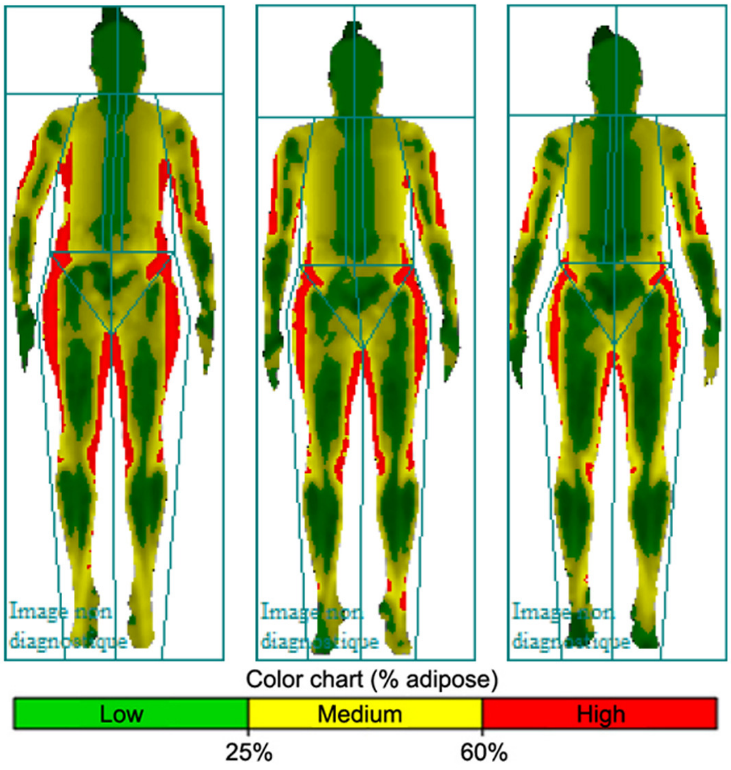

Figure. Dual X-ray absorptiometric scanning before (left) the cryotherapy procedure, and then 14 (middle) and 60 days (right) after 5 procedures, illustrating long-term loss of adipose tissue.

Although such examples suggest that adipose tissue loss may continue for an extended period after the cryotherapy procedure, it was not possible to supervise these subjects for potential changes in activity/exercise and/or caloric intake during the follow-up period, and for this reason the enduring changes may not unambiguously ascribed to metabolic changes induced by the cryotherapy procedure.

References

1 Gayoso-Diz *et al.* (2013) Insulin resistance (HOMA-IR) cut-off values and the metabolic syndrome in a general adult population: effect of gender and age: EPIRCE cross-sectional study. *BMC Endocrine Disorders* 13, 47

2 Hall, K.D. (2008) What is the required energy deficit per unit weight loss? *Int. J. Obes.* 32, 573-576

3 Thomas, D.M. *et al.* (2013) Can a weight loss of one pound a week be achieved with a 3500-kcal deficit? Commentary on a commonly accepted rule. *Int. J. Obes.* 37, 1611-1613

4 Hall, K.D. and Chow, C.C. (2013) Why is the 3500 kcal per pound weight loss rule wrong? *Int. J. Obes.* 37, 1614

5 Zelickson, B. *et al.* (2009) Cryolipolysis for noninvasive fat cell destruction: initial results from a pig model. *Dermatol. Surg.* 35, 1462-1470

6 Manstein, D. *et al.* (2008) Selective cryolysis: a novel method of non-invasive fat removal. *Lasers Surg. Med.* 40, 595-604
